# Maternal and umbilical cord plasma concentrations of antiseizure medications: Results from the observational MONEAD study

**DOI:** 10.1002/epi.70129

**Published:** 2026-05-28

**Authors:** Charul Avachat, Kimford J. Meador, Page B. Pennell, Angela K. Birnbaum, MONEAD Investigator Group, Delmaris Acosta‐Cotte, Sandra Alhaj, Stephanie Allien, Taimur Anwar, Anto Bagic, Gregory L. Barkley, Donald Bearden, Susan Beers, Irena Bellinski, Christin Bermudez, Kristina Blessing, Katrina Boyer, Camilla Casadei, Patricia Chang, Li Chen, Andrea Cheng‐Hakimian, Melanie Choe, Kirsten Cleary, Tobi Clements, Joseph Coda, Pam Coe, Jules Constantinou, Yael Cukier Cukier, Danielle Culbreth, Elizabeth Cunningham, Kayla Darris, Lisa Davis, Rosemarie Delucca, Jennifer DeWolfe, Jessica Dimos, Mary Dolan, Maurice Druzin, Joyce Echo, Sarah Ellis, Pedro Figueredo, Richard Finnell, Kellie Flood‐Schaffer, Jacqueline French, Mark Friedman, Shailaja Gaddam, Satya Gedela, Elizabeth Garard, Christine Ghilian, William Grobman, Cheryl Hall, Ellen Hanson, Jacqueline Helcer Helcer, Paige Hickey, Gregory Holmes, Theresa Holmes, Dominic Ippolito, George Jewell, Arundhathi Jeyabalan, Emily Johnson, Michelle Kim, Gregory Krauss, Casey Krueger, David Labiner, Hadley Lange, Erin Latif, Connie Lau Lau, Shari Lawson, Brenda Leung, William MacAllister, James Maciulla, Hayley Madeiros, Nazin Mahmood, Jennie Mao, Ryan May, Paul McCabe, Frederick T. McElrath, Erica Meltzer Meltzer, Lucy Mendoza, Emily Miller, John W. Miller, Michelle Miranda, Jennifer Moon, Eugene Moore, Melissa Morris, Chris Morrison, Lorene Nelson, Melanee Newman, Alisha Olson, Kim Ono, John Owen, Alison Pack, Michael Paglia, Yong Park, Lamar Parker, Christina Patterson, Sonia Perez, Jenny Pohlman, Alison Pritchard, Michael Privitera, Krestin Radonovich, Patty Ray, Katie Reger, Gustavo Rey, Matthew Ryan, Yasin Salih, Carla Sandles, William Schweizer, Jordan Seliger, Enrique Serrano, Nilay Shah, Elizabeth Shashkova, Traci Sheer, Yvonne Sheldon, Rachel Sierra, Marianna Spanaki‐Varelas, Anna Steele, Jennifer Steele, Alice Stek, Zachary Stowe, Jolie Strauss, Suzanne Strickland, Melissa Sutcliffe, Hima Bindu Tam Tam, Diane Teagarden, Andrea Thomas, Matthew Thompson, Jeffery Tsai, Alexandra Urban, Linda Van Marter, Naymee Velez‐Ruiz, Yue Wang, Vibhangini Wasade, Taylor Weinau, Peter Wells, Carrie Wiles, Mark Yerby, Amy Young, Andrew Zillgitt, Annette Zygmunt

**Affiliations:** ^1^ Department of Experimental and Clinical Pharmacology, College of Pharmacy University of Minnesota Minneapolis Minnesota USA; ^2^ Department of Neurology & Neurological Sciences Stanford University School of Medicine Palo Alto California USA; ^3^ Department of Neurology University of Pittsburgh School of Medicine Pittsburgh Pennsylvania USA; ^4^ Present address: Bristol Myers Squibb Princeton NJ USA

**Keywords:** antiseizure medications, cord blood, fetus, maternal, umbilical cord

## Abstract

**Objective:**

Unanticipated changes in antiseizure medication (ASM) exposure can lead to subtherapeutic or toxic medication concentrations in the mother and unnecessary drug exposure for the fetus. The objectives of this study were to characterize ASM concentrations in mother's and cord blood at delivery in women with epilepsy (PWWE).

**Methods:**

The analysis included PWWE (aged 14–45 years) from the Maternal Outcomes and Neurodevelopmental Effects of Antiepileptic Drugs study who were on ASM monotherapy or polytherapy at delivery. Women with an intelligence quotient < 70, major medical illness, substance use, and poor treatment adherence were excluded. Blood samples were collected from the mother and umbilical cord on the day of delivery. The ratio of umbilical cord to maternal plasma concentration at birth was used as a measure of fetal in utero exposure. Pearson correlation coefficients were calculated to determine the degree of correlation between ASM maternal and cord plasma concentrations. A *p*‐value of <.05 was deemed to be significant.

**Results:**

Data from 207 infants included eight pairs of twins (104 females and 103 males) born to 199 women (140 on monotherapy and 59 on polytherapy), yielding 305 paired maternal and umbilical cord ASM concentrations. Significant correlation was observed between umbilical cord and maternal plasma concentrations for total and unbound carbamazepine, total and unbound carbamazepine‐10,11‐epoxide, gabapentin, lamotrigine, levetiracetam, total and unbound oxcarbazepine, and zonisamide. The mean ratios (SD) of umbilical cord plasma concentration to maternal plasma concentration were as follows: carbamazepine = .84 (.18), unbound carbamazepine = 1.15 (.47), carbamazepine‐10,11‐epoxide = .87 (.18), unbound carbamazepine epoxide = 1.17 (.36), gabapentin = 1.56 (.28), lacosamide = .96 (.29), levetiracetam = 1.1 (.53), lamotrigine = .92 (.26), oxcarbazepine = 1.01 (.22), unbound oxcarbazepine = 1.1 (.46), topiramate = .98 (.38), valproic acid = 1.03 (.85), unbound valproic acid = .73 (.74), and zonisamide = .93 (.12).

**Significance:**

Umbilical cord to maternal ASM concentration ratios were close to 1.0, indicating placental passage of ASMs. Gabapentin had the highest ratio, suggesting possible accumulation of drug. Additional studies are needed for infrequently prescribed ASMs.

**Key points**

- Maternal and cord blood concentrations were correlated.
- The ratios of umbilical cord to maternal ASM concentrations vary among ASMs, but most were close to unity.
- Gabapentin had the highest umbilical cord to maternal ASM concentration ratio (>1.0), suggesting possible accumulation of drug.

## 1 INTRODUCTION

A fundamental aspect of antiseizure medication (ASM) management is to maintain adequate circulating blood drug concentrations to reduce seizures, thus decreasing potential side effects. As pregnancy can substantially alter the pharmacokinetics of some ASMs; dose adjustments, including substantial dose increases may be necessary and appropriate to maintain the individual's prepregnancy target concentration and prevent breakthrough seizures.^1^ High doses of certain ASMs have been associated with major birth defects, including congenital heart disease, cleft lip/palate, urogenital defects, and neural tube defects.^2^, ^3^, ^4^, ^5^, ^6^, ^7^, ^8^ Additionally, for several ASMs these effects are dose dependent.^9^ Other adverse outcomes in neonates such as premature delivery, small for gestational age birthweight, low Apgar scores, impaired cognition, and abnormal behaviors have also been reported.^2^ Thus, dose adjustments need to balance seizure control for the mother with minimal ASM exposure for the developing fetus via placental transport of drugs. Therefore, therapeutic drug monitoring is often employed to determine optimal ASM management during pregnancy.^10^

The placenta is a major link between the mother and the baby, transferring not only nutrients and oxygen but also any xenobiotics taken by the mother. The degree of transplacental passage of drugs is dependent on several gestational week factors, including mechanism of transfer (active vs. passive diffusion), influence of placental transporters, presence of metabolizing enzymes in the placenta, molecular weight of the drug, drug binding to proteins, lipophilicity of the drug, and extent of drug dissociation.^11^

Measuring circulating fetal drug concentrations during pregnancy is not possible; however, umbilical cord ASM blood concentrations can be obtained to estimate ASM exposure of the fetus. Maternal drug concentration is the primary factor that determines umbilical cord concentration, which in turn largely relies on the dose of ASM taken by the mother. The ratio of umbilical to maternal blood/plasma concentration acts as a surrogate for fetal drug exposure, where a high level of correlation between maternal and umbilical cord blood concentrations is indicative of substantial drug transfer through the placenta.^12^, ^13^, ^14^ The objective of this study was to characterize ASM concentrations in maternal and umbilical cord blood at delivery in mothers with epilepsy who were enrolled in the 20‐site Maternal Outcomes and Neurodevelopmental Effects of Antiepileptic Drugs (MONEAD) study.

## 2 MATERIALS AND METHODS

### 2.1 Study design

The MONEAD study is a prospective, observational, multisite study that enrolled women with epilepsy during the pregnancy and postpartum period as well as the children born to these women. The study was approved by the institutional review boards at each of the 20 participating sites. Enrollment in the study occurred from December 19, 2012 through February 11, 2016. Participants were expected to attend a total of seven visits, comprising three visits during pregnancy, one birth visit, and three postpartum visits. For the present analysis, pregnant women with epilepsy, between the ages of 14 and 45 years, receiving ASM monotherapy or polytherapy during the birth visit were included. Women with an intelligence quotient (IQ) < 70, major medical illness, substance use, poor treatment adherence, and ≤20 gestational weeks were excluded. Seizures, ASM dosing, and adherence were tracked via an electronic app, with dosing being verified at each study visit. Informed consent was obtained from all the women participating in the study. Data were analyzed from May 1, 2014 to December 30, 2023.

### 2.2 Sample collection and bioanalysis

The analysis used blood samples collected from the mother's and cord blood at the birth visit. The plasma was separated, and samples were frozen at −80°C within 2 h after collection. Cord blood was collected using aseptic collection techniques. Samples were batched and shipped to the MONEAD Pharmacokinetic Core Laboratory (University of Minnesota). All ASM concentrations were analyzed using liquid chromatography–mass spectrometry assays validated in the Pharmacokinetics Core Laboratory, measuring multiple ASMs simultaneously.^15^ ASMs measured were carbamazepine, gabapentin, lacosamide, levetiracetam, lamotrigine, oxcarbazepine, topiramate, pregabalin, phenobarbital, phenytoin, valproic acid, and zonisamide. The quantitation range for ASMs is described in Table S1. For women receiving carbamazepine, the concentration of unbound carbamazepine, its metabolite (carbamazepine‐10,11‐epoxide), and carbamazepine‐10,11‐epoxide unbound were measured in maternal and cord blood plasma. For women receiving oxcarbazepine, phenytoin, and valproic acid, the concentration of unbound oxcarbazepine, unbound phenytoin, and unbound valproic acid was also measured. Multiple mother–cord blood pairs were available in certain cases; some women who were on polytherapy, and thus more than one ASM, was also included in the analysis. Data collected included information on time and date of dose received by the mother, and time and date of sample collection from the mother and cord blood. Demographic information collected on mothers included height, weight, age, race, ethnicity, and gestational week of delivery.

### 2.3 Statistical analysis

Our primary goal was to determine the degree of correlation between maternal and cord plasma ASM concentrations. For this purpose, the number of maternal–cord blood pairs was identified, and descriptive statistics were performed. Continuous variables, namely age, gestational weeks, and maternal and cord blood concentrations, were described using median and range. Categorical variables like race and ethnicity were expressed as number of individuals belonging to a particular category. To determine the degree of correlation between ASM maternal and cord plasma concentrations, Pearson correlation coefficients were calculated. A *p*‐value of <.05 was deemed to be statistically significant. The ratio of umbilical cord to maternal plasma concentration was determined for all the ASMs (bound and unbound) and their metabolites, was used as a measure of fetal in utero exposure, and is reported as the mean (SD) to enable comparison to previous literature values. Among patients receiving levetiracetam, two women had cord to maternal plasma concentration ratios of .023 and 14.9, respectively, which was more than ±3 SD away from the mean, and were thus considered to be outliers and excluded. Statistical analyses were performed in R (version 4.0.3).

## 3 RESULTS

### 3.1 Patients

After excluding mothers with missing concentrations and removing outliers, concentrations in 199 women (140 on monotherapy and 59 on polytherapy) giving birth to 207 infants were available. Three hundred five matched pairs of maternal and umbilical cord ASM concentrations remained: 12 carbamazepine, 12 carbamazepine epoxide, 12 unbound carbamazepine, 12 carbamazepine epoxide unbound, 3 gabapentin, 5 lacosamide, 95 levetiracetam, 103 lamotrigine, 11 oxcarbazepine, 11 unbound oxcarbazepine, 2 phenobarbital, 1 pregabalin, 1 phenytoin, 1 unbound phenytoin, 4 topiramate, 3 valproic acid, 3 unbound valproic acid, and 14 zonisamide. The distribution of ASMs reflects usage at that time in PWWE at the 20 tertiary centers. Only four women among those on polytherapy were receiving potentially interacting ASM combinations of lamotrigine + phenobarbital, lamotrigine + oxcarbazepine + valproic acid, and lamotrigine + carbamazepine. Data from 207 live infants (104 female and 103 male) included eight pairs of twins. The mean (SD) infant birthweight was 3.16 kg (.49) for females and 3.39 kg (.56) for males. The median age and range were comparable across different ASM categories (Table 1). Gestational age at the time of birth was also similar across different ASMs. A majority of women (86.4%) identified as White, and 82.4% belonged to a non‐Hispanic ethnicity.

**TABLE 1:** Demographic characteristics of women stratified by ASM.

ASM	CBZ	GBP	LCM	LEV	LTG	OXC	TPM	VPA	ZNS
*n* ^a^	12	3	5	91	100	9	4	3	13
Age, years, median (range)	30 (21–40)	33 (27–39)	35 (20–41)	32 (20–47)	32 (20–44)	31 (20–33)	25.5 (24–29)	32 (28– 34)	27.5 (20–37)
Gestational weeks at delivery, median (range)	39 (35–41)	37 (36–40)	38 (36–39)	39 (33–41)	39 (33–41)	39 (37–40)	39 (38–41)	39 (39– 40)	39 (34–41)
Race, *n*									
White	7	3	3	81	88	8	3	3	13
African American	NA	NA	1	2	4	1	NA	NA	NA
Asian	2	NA	1	2	3	NA	1	NA	NA
Multiracial	1	NA	NA	4	3	NA	NA	NA	NA
Other or unknown	2	NA	NA	2	2	NA	NA	NA	NA
Ethnicity, *n*									
Hispanic	4	NA	NA	23	12	2	1	NA	3
Non‐Hispanic	8	3	5	68	88	7	3	3	10

Abbreviations: ASM, antiseizure medication; CBZ, carbamazepine; GBP, gabapentin; LCM, lacosamide; LEV, levetiracetam; LTG, lamotrigine; *n*, number of women receiving the ASM; NA, not applicable; OXC, oxcarbazepine; TPM, topiramate; VPA, valproic acid; ZNS, zonisamide.

^a^ Includes women receiving both monotherapy and polytherapy. *n* may not add up to total number of individuals in the study, because some women were receiving polytherapy and are thus represented in two or more drug categories.

### 3.2 Correlation between umbilical cord and maternal ASM concentration

**FIGURE 1:**
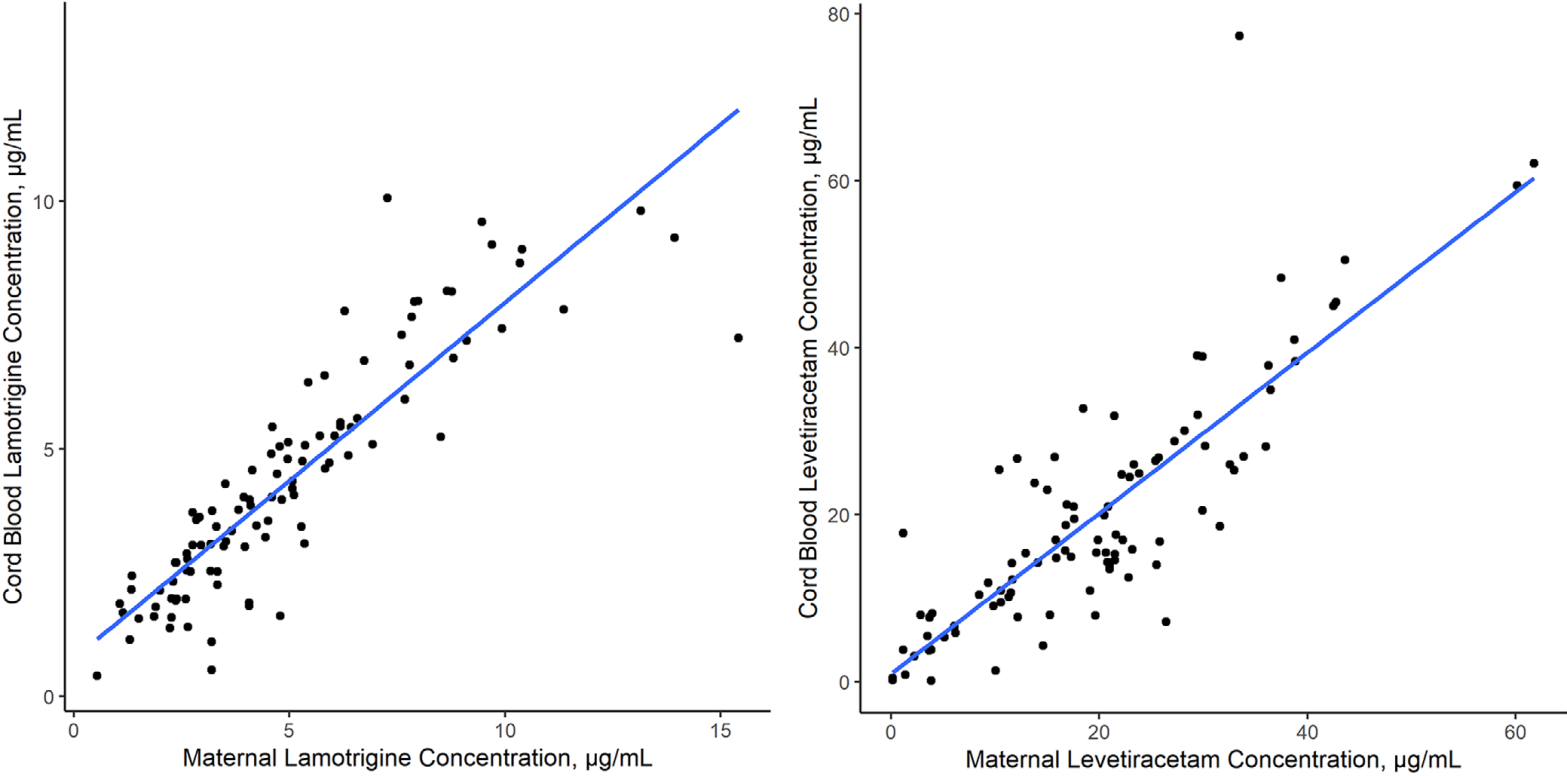
Associations of maternal and umbilical cord drug concentrations for levetiracetam and lamotrigine. Black dots represent individual observations and the blue line represents the line of best fit.

A significant correlation (*p* < .05) was observed between umbilical cord and maternal plasma concentrations for carbamazepine, carbamazepine epoxide, unbound carbamazepine, carbamazepine epoxide unbound, lamotrigine, levetiracetam, gabapentin, oxcarbazepine, unbound oxcarbazepine, and zonisamide (Table 2), which constituted 93.4% pairs of umbilical cord to maternal plasma concentrations. Correlation plots for ASMs are presented in Figures 1 and s1.

**TABLE 2:** Pearson and Spearman correlation coefficients between maternal and umbilical cord ASM concentrations.

ASM/metabolite	Number of pairs	Pearson correlation coefficient (*p*)	Literature value of CPC/MPC, mean^a^	Current study value of CPC/MPC, mean
Carbamazepine	12	.63 (.029)	.78 (*n* = 75)^16^	.84
Carbamazepine epoxide	12	.97 (<.0001)	.79 (*n* = 64)^16,17^	.87
Carbamazepine unbound	12	.81 (.0013)	1.42 (*n* = 9)^17^	1.15
Carbamazepine epoxide unbound	12	.91 (<.0001)	–	1.17
Gabapentin	3	1.0 (.021)	1.7 (*n* = 6)^26^	1.56
Lacosamide	5	.65 (.23)	1.03 (*n* = 1)^25^	.96
Lamotrigine	103	.89 (<.0001)	.92 (*n* = 40)^23^	.92
Levetiracetam	95	.84 (<0.0001)	1.1 (*n* = 14)^20^	1.1
Oxcarbazepine	11	.98 (<.0001)	1.11 (*n* = 3)^17^	1.01
Oxcarbazepine unbound	11	.98 (<.0001)	–	1.1
Topiramate	4	.85 (.15)	.93 (*n* = 11)^28^	.98
Valproic acid	3	.75 (.46)	1.59 (*n* = 8)^30^	1.03
Valproic acid unbound	3	.29 (.81)	.5 (*n* = 8)^30^	.73
Zonisamide	14	.99 (<.0001)	.92 (*n* = 1)^24^	.93

Abbreviations: ASM, antiseizure medication; CPC, cord plasma concentration; MPC, maternal plasma concentration; *n*, number of participants.

^a^ Literature values from studies with highest number of participants reported.

### 3.3 Ratio of umbilical cord to maternal ASM concentrations

**FIGURE 2:**
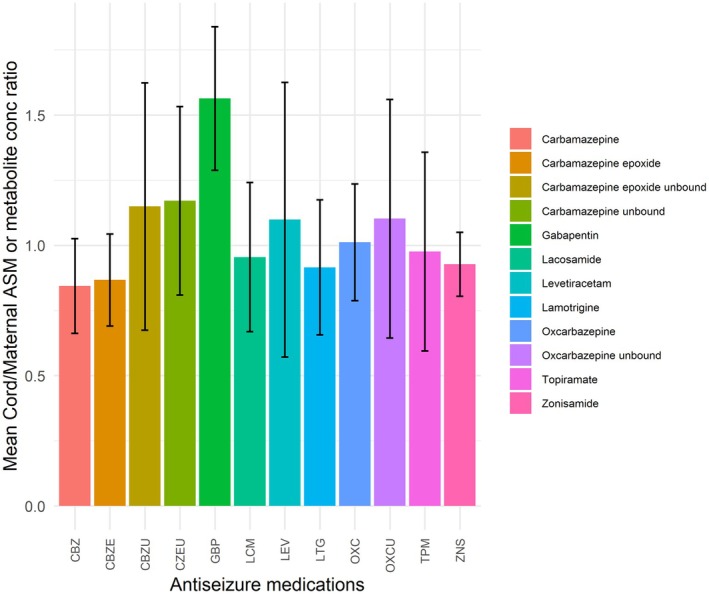
Ratio of cord to maternal antiseizure medication (ASM)/metabolite concentration. Bars represent the mean ratios by ASM. Error bars represent standard errors. Abbreviations: CBZ, carbamazepine; CBZE, carbamazepine epoxide; CBZU, carbamazepine epoxide unbound; CZEU, carbamazepine unbound; GBPC, gabapentin; LCMC, lacosamide; LEVC, levetiracetam; LTGC, lamotrigine; OXCC, oxcarbazepine; OXCU, oxcarbazepine unbound; TPMC, topiramate; ZNSC, zonisamide.

The mean ratio of umbilical cord to maternal plasma concentrations of all ASMs other than gabapentin was close to unity (i.e., 1.0), showing equivalent umbilical cord ASM concentrations. The ratio of mean (SD) umbilical cord to maternal plasma concentration for ASMs was carbamazepine = .84 (.18), unbound carbamazepine = 1.15 (.47), carbamazepine epoxide = .87 (.18), unbound carbamazepine epoxide = 1.17 (.36), gabapentin = 1.56 (.28), lacosamide = .96 (.29), levetiracetam = 1.1 (.53), lamotrigine = .92 (.26), oxcarbazepine = 1.01 (.22), unbound oxcarbazepine = 1.1 (.46), topiramate = .98 (.38), valproic acid = 1.03 (.85), unbound valproic acid = .73 (.74), and zonisamide = .93 (.12) (Table 3, Figure 2). A comparison between literature and our study values of cord to maternal drug concentration ratios is presented in Table 2.

**TABLE 3:** Maternal and cord plasma concentrations and ratios for different ASMs.

	CBZ	CBZE	CBZU	CBZEU	GBP	LCM	LEV	LTG	OXC	OXCU	TPM	VPA	VPAU	ZNS
*n*	12	12	12	12	3	5	95	103	11	11	4	3	3	14
Median CPC, μg/mL	5.1	1.5	.8	.5	2.9	5.5	15.8	3.9	6.7	3.8	2.8	28.3	2	8.1
Range of CPC, μg/mL	2.7–6.4	.8–3.3	.1–1.4	.2–1.0	2.2–10.3	2.5–9.6	.2–77.3	.4–10.1	3.7–24.2	1.8–13.7	2.1–4.7	3.1–62.1	.2–6.5	1.5–27.8
Median MPC, μg/mL	5.7	1.6	.8	.4	1.7	7.5	19.6	4.5	8.5	4.1	2.9	26.3	8.8	9.1
Range of MPC, μg/mL	3.4–7.9	.6–4.4	.2–1.6	.1–1.1	1.3–8.3	2.2–9.4	.2–61.8	.5–13.9	3.0–26.3	1.1–15.8	2–6.3	15‐7–52.5	1.4–16.6	1.9–23.7
Median ratio of CPC/MPC	.82	.85	1.08	.98	1.69	1.07	1.03	.9	.97	.94	.81	1.18	.74	.93
Range of CPC/MPC ratio	.6–1.2	.6–1.2	.8–2	.6–2	1.3–1.8	.4–1.1	.1–3.2	.2–1.8	.7–1.5	.8–2.4	.7–1.5	.1–1.8	.01–1.43	.7–1.2

Abbreviations: ASM, antiseizure medication; CBZ, carbamazepine; CBZE, carbamazepine epoxide; CBZEU, carbamazepine epoxide unbound; CBZU, unbound carbamazepine; CPC, cord plasma concentration; GBP, gabapentin; LCM, lacosamide; LEV, levetiracetam; LTG, lamotrigine; MPC, maternal plasma concentration; *n*, number of pairs; OXC, oxcarbazepine; OXCU, unbound oxcarbazepine; TPM, topiramate; VPA, valproic acid; VPAU, unbound valproic acid; ZNS, zonisamide.

## 4 DISCUSSION

The ratio of umbilical cord to maternal ASM concentrations can provide valuable information regarding the extent of drug exposure of the fetus. A significant correlation between maternal and umbilical plasma concentrations was observed for all ASMs except lacosamide, valproic acid, and topiramate, ASMs with the smallest number of pairs available for correlation analysis. Our study is the first to provide information for possible fetal exposure of unbound carbamazepine epoxide and unbound oxcarbazepine. Our study adds substantially to the prior literature, which had only case studies with single individuals for lacosamide and zonisamide. The mean ratios of ASMs/metabolites in our study ranged from .84 for carbamazepine to 1.69 for gabapentin, indicating significant placental passage, resulting in substantial fetal exposure.

In individuals receiving carbamazepine monotherapy or carbamazepine + noninteracting ASMs, previously reported ratios of carbamazepine and its epoxide metabolite were .78 and .79, respectively, which are slightly lower than the values observed in our study.^16^, ^17^ Unbound carbamazepine and its unbound epoxide metabolite had a higher ratio in comparison to the total drug and metabolite. This higher ratio may be due to complete transplacental passage of the free drug.

In individuals receiving levetiracetam, the mean ratio was similar to that of literature values.^17^, ^18^, ^19^, ^20^ Our study included 95 pairs of maternal–umbilical cord concentrations from patients on levetiracetam, representing the most extensive sample size reported in the existing literature. The levetiracetam ratios ranged from .1 to 3.2, wider than those reported in the literature, and may be more reflective of the true interpatient variability in pregnant patients taking levetiracetam.^17^, ^18^, ^19^, ^20^ High levetiracetam concentrations have been shown to affect the expression of several transporter‐related genes in human placental cells.^21^ Additionally, a negative exposure‐dependent association of levetiracetam with a verbal index score (that measures an individual's verbal knowledge and reasoning abilities) and key behavior outcome Adaptive Behavior Assessment System General Adaptive Composite (ABAS‐3 GAC) (which evaluates adaptive skills that an individual uses to function independently) has been reported in a recent study. Based on these findings, caution should be exercised when escalating the dose of levetiracetam during pregnancy.^22^

The ratios of lamotrigine concentrations observed in our study with the larger dataset encompassing 102 matched maternal–umbilical cord pairs align consistently with those documented in the existingliterature.^17^, ^23^ Total and oxcarbazepine ratios were 1.01 and 1.11, respectively. The current investigation represents the most extensive cohort of patients undergoing zonisamide treatment, demonstrating a mean ratio of .93, consistent with the ratio observed in the case study subject.^24^ Lacosamide, a comparatively new ASM, exhibited a mean ratio of .96 (*n* = 5) in our study. This ratio is similar to the value reported in a case study involving a single individual taking lacosamide, which was 1.03. Regardless of the limited number of patients prescribed gabapentin in both current and previous studies, a consistently elevated umbilical cord to maternal ratio was noted, potentially attributed to gabapentin accumulation facilitated by placental L‐type amino acid transporter 1 (LAT1/OCTN1) transporters.^25^, ^26^, ^27^ Mean topiramate ratios were also similar to what has been reported earlier.^28^ Valproic acid unbound concentrations in the cord blood was markedly lower that the total drug concentration. Despite this low placental passage, fetal valproic acid exposure has been shown to negatively impact the IQ scores of these children in comparison to drugs such as carbamazepine, lamotrigine, and phenytoin in a dose‐dependent manner.^7^ In our study, a high variability in valproic acid concentrations was observed possibly due to a lower sample size.

There are certain limitations to our study. Cord blood collection can represent a mixture of maternal and infant blood and may not be reflective of only fetal/infant drug exposure; however, it can be useful in identifying drugs that may accumulate in the fetal compartment. The number of individuals on certain ASMs other than lamotrigine and levetiracetam is small, because they are not as commonly prescribed to pregnant women with epilepsy. Thus, larger sample size studies in individuals on less frequently prescribed ASMs and on newer ASMs approved by the US Food and Drug administration in the past decade need to be conducted.

## 5 CONCLUSIONS

This study adds substantially to the literature and provides information on the exposure to ASMs of the fetus during pregnancy in women with epilepsy. The ratio of umbilical cord to maternal ASM concentrations vary among ASMs, but most were close to 1.0, indicating complete placental passage of ASMs. For gabapentin, irrespective of the small sample size, the mean umbilical cord to maternal ASM concentration ratio was greater than 1.0, signifying a possible accumulation of the drug in the fetal compartment. In the future, these data will be paired with infant and child outcome data in MONEAD to assess the potential impact of maternal drug exposure on the health and development of the child. Future studies employed to separate maternal and fetal blood will enhance our ability to accurately analyze the partitioning of the ASMs across the placenta, leading to more precise medical interventions and improved outcomes for both mother and baby.

## AUTHOR CONTRIBUTIONS

Charul Avachat and Angela K. Birnbaum were involved with the analysis and interpretation of data and drafting/revision of the manuscript. Page B. Pennell, Kimford J. Meador, and Angela K. Birnbaum were involved with study design, acquisition of data, analysis and interpretation of data, and drafting/revision of the manuscript.

## FUNDING INFORMATION

This work was supported by the National Institute of Neurological Disorders and Stroke and National Institute of Child Health and Development (grants U01‐NS038455 [K.J.M., P.B.P.], 2U01‐NS038455 [K.J.M., P.B.P.], and R01‐HD105305 [A.K.B., P.B.P.]) and the University of Minnesota’s Doctoral Dissertation Fellowship (C.A.). The funding organizations did not directly participate in the design and conduct of the study; collection, management, analysis, and interpretation of the data; preparation, review, or approval of the manuscript; and decision to submit the manuscript for publication.

## CONFLICT OF INTEREST STATEMENT

C.A. has no conflicts to disclose. K.J.M. has received research support from the National Institutes of Health (NIH), Eisai, and Medtronic; the Epilepsy Study Consortium pays K.J.M.’s university for his research consultant time related to Eisai, GW Pharmaceuticals, NeuroPace, Novartis, Supernus, Upsher‐Smith Laboratories, UCB Pharma, Vivus Pharmaceuticals, and Xenon Pharmaceuticals. P.B.P. has received royalties as a contributing author for UpToDate, research support from NIH, honoraria for grant reviews from NIH, and honoraria and travel reimbursements for continuing medical education presentations from academic medical centers and professional societies. A.K.B. has received research support from the NIH, Vireo Health, and UCB Pharma. A.K.B. is also a coinventor of a patent for intravenous carbamazepine (Lundbeck Pharmaceuticals). We confirm that we have read the Journal’s position on issues involved in ethical publication and affirm that this report is consistent with those guidelines.

## Supporting information


**TABLE S1** Assay quantitation range for antiseizure medications.

**FIGURE S1** Associations of maternal and umbilical cord drug concentrations.

**TABLE S1** epi70129‐sup‐0003‐DataS1.pdf.

## Data Availability

Research data are not shared.
