# Supplementary material for: Maternal and umbilical cord plasma concentrations of antiseizure medications: Results from the observational MONEAD study
Source: Epilepsia. 2026 May 28;67(7):3401–9. doi: 10.1002/epi.70129 (PMC13361046; doi:10.1002/epi.70129)
Supplement: Supplementary file 1 — TABLE S1 Assay quantitation range for antiseizure medications. [file EPI-67-3401-s002.docx]

**Supplementary Table 1** Assay quantitation range for antiseizure medications

| **Drugs** | | **Quant. Range (ug/mL)** |
| --- | --- | --- |
| *LEV* | Levetiracetam | 0.1 - 80 |
| *GBP* | Gabapentin | 0.1 - 20 |
| *PGB* | Pregabalin | 0.1 - 20 |
| *LCM* | Lacosamide | 0.1 - 80 |
| *LTG* | Lamotrigine | 0.3 - 25 |
| *CBZ* | Carbamazepine | 0.3 - 25 |
| *PHT* | Phenytoin | 0.5 - 40 |
| *TPM* | Topiramate | 0.4 - 30 |
| *OXC* | Oxcarbazepine | 0.5 - 40 |
| *ZNS* | Zonisamide | 0.6 - 50 |
| *PB* | Phenobarbital | 0.5 - 40 |
| *VPA* | Valproic Acid | 0.5 - 500 |
