# Supplementary figures and images for: Maternal and umbilical cord plasma concentrations of antiseizure medications: Results from the observational MONEAD study
Source: Epilepsia. 2026 May 28;67(7):3401–9. doi: 10.1002/epi.70129 (PMC13361046; doi:10.1002/epi.70129)

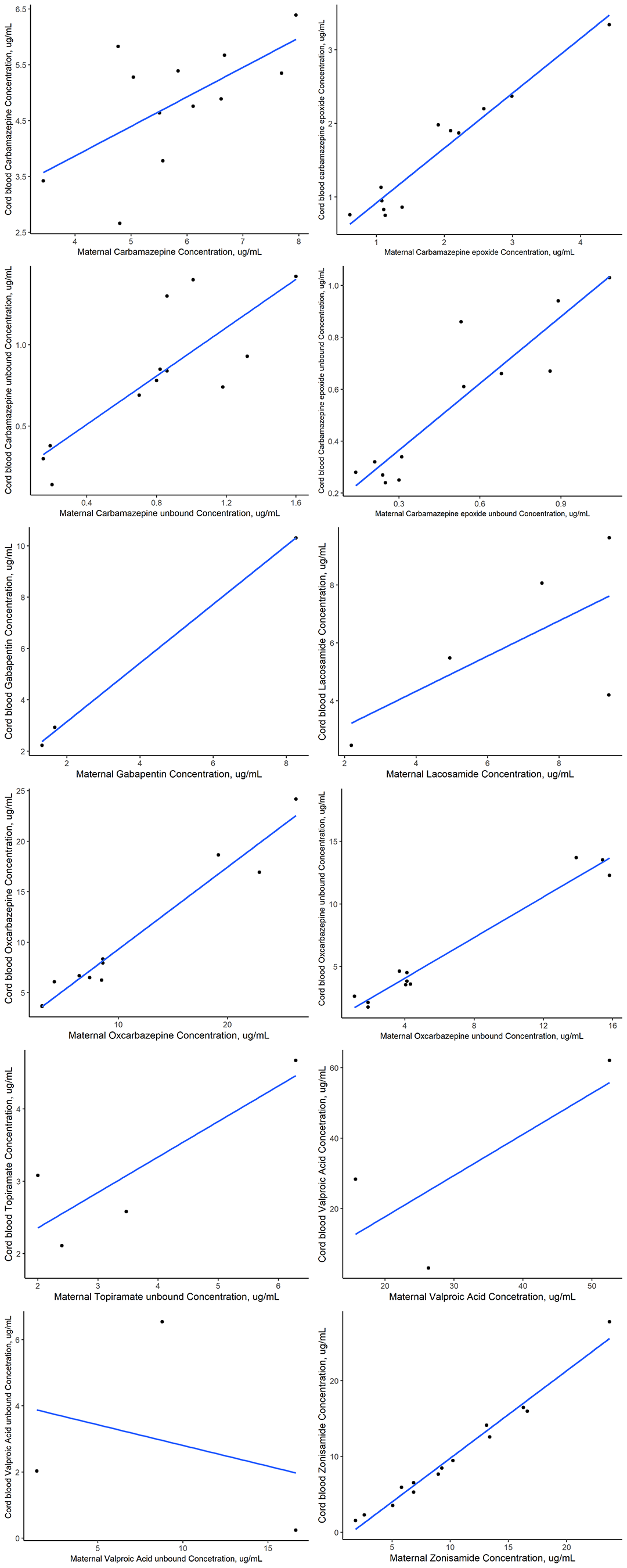

Supplement: Supplementary file 2 — FIGURE S1 Associations of maternal and umbilical cord drug concentrations. [file EPI-67-3401-s003.tiff]
