# Supplementary material for: Maternal and umbilical cord plasma concentrations of antiseizure medications: Results from the observational MONEAD study
Source: Epilepsia. 2026 May 28;67(7):3401–9. doi: 10.1002/epi.70129 (PMC13361046; doi:10.1002/epi.70129)
Supplement: Supplementary file 3 — TABLE S1 epi70129‐sup‐0003‐DataS1.pdf. [file EPI-67-3401-s001.pdf]

Supplemental Online Content: Nonauthor Collaborators

*Indicates required information. Only first name, last name, and suffix will appear in PubMed.

**Group Name(s):* MONEAD Investigator Group**					
Delmaris	Acosta-Cotte		MA	Augusta University	Site Neuropsychologist
Sandra	Alhaj		BS	University of Pittsburgh	Research Assistant
Stephanie	Allien		PAC	Brigham & Women's Hospital	Research Physician's Assistant
Taimur	Anwar		MD	Henry Ford Hospital	OB Co-Investigator
Anto	Bagic		MD	University of Pittsburgh	Site PI
Gregory L.	Barkley		MD	Henry Ford Hospital	Site PI
Donald	Bearden		PhD	Emory University Children's Healthcare	Site Neuropsychologist
Susan	Beers		PhD	University of Pittsburgh	Site Neuropsychologist
Irena	Bellinski		RN	Northwestern University	Research Assistant
Christin	Bermudez		PhD	University of Miami	Site Psychometrist
Kristina	Blessing		MSW	Geisinger Clinic	Research Assistant
Katrina	Boyer		PhD	Boston Children's Hospital	Site Neuropsychologist
Camilla	Casadei		BA	Columbia University	Research Assistant
Patricia	Chang		MD	Minnesota Epilepsy Group	OB co-Investigator
Li	Chen		BA	Brigham & Women's Hospital	Research Assistant
Andrea	Cheng-Hakimian		MD	University of Washington	Co-Investigator
Melanie	Choe		MS	Brigham & Women's Hospital	Research Assistant
Kirsten	Cleary		MD	Columbia University	OB co-Investigator
Tobi	Clements		BA	New York University	Research Assistant
Joseph	Coda		PsyD	Northwestern University	Site Neuropsychologist
Pam	Coe		MS	John Hopkins University	Research Assistant
Jules	Constantinou		MD	Henry Ford Hospital	Co-Investigator
Yael Cukier	Cukier		PhD	Northwell Health	Site Neuropsychologist
Danielle	Culbreth		MA		Project Traveling Neuropsychologist
Elizabeth	Cunningham		MPH	Northwestern University	Research Assistant
Kayla	Darris		BA	University of Arizona	Research Assistant
Lisa	Davis		BA	The Emmes Company	Administrative Coordinator
Rosemarie	Delucca		RN	Geisinger Clinic	Research Assistant
Jennifer	DeWolfe		DO	University of Alabama at Birmingham	Site PI
Jessica	Dimos		BS	Wake Forest University Health Sciences	Research Assistant
Mary	Dolan		MD	Emory University	OB co-Investigator
Maurice	Druzin		MD	Stanford University	Co-Director of OB Core
Joyce	Echo		PhD	Columbia University	Site Neuropsychologist
Sarah	Ellis		BS	Minnesota Epilepsy Group	Research Assistant
Pedro	Figueredo		MD	University of Miami	Research Assistant
Richard	Finnell		PhD	Baylor College of Medicine	Consultant on Teratogenicity
Kellie	Flood-Schaffer		MD	University of Cincinnati	OB Co-Investigator
Jacqueline	French		MD	NYU Grossman School of Medicine	
Mark	Friedman		MS	The Emmes Company	Clinical Systems Analyst
Shailaja	Gaddam		MD	Henry Ford Hospital	Co-Investigator
Satya	Gedela		MD	University of Pittsburgh	Co-Investigator
Elizabeth	Garard		MD	Northwestern University	
Christine	Ghilian		PhD	Emory University Children's Healthcare	Site Neuropsychologist
William	Grobman		MD	Northwestern University	OB Co-Investigator
Cheryl	Hall		LPN	University of Alabama at Birmingham	Research Assistant
Ellen	Hanson		PhD	Boston Children's Hospital	Site Neuropsychologist
Jacqueline Helcer	Helcer		PhD	Northwell Health	Site Neuropsychologist
Paige	Hickey		BS	Boston Children's Hospital	Site Psychometrist
Gregory	Holmes		MD	University of Vermont	Consultant and Executive Committee Member
Theresa	Holmes		BA	Henry Ford Hospital	Research Assistant
Dominic	Ippolito		MS	The Emmes Company	Project Manager
George	Jewell		PhD	University of Cincinnati	Site Neuropsychologist
Arundhathi	Jeyabalan		MD	University of Pittsburgh	OB Co-Investigator
Emily	Johnson		MD	Johns Hopkins University	Site PI
Michelle	Kim		PhD	University of Washington	Site Neuropsychologist
Gregory	Krauss		MD	Johns Hopkins University priorSite PI	Prior Site PI
Casey	Krueger		PhD	Stanford Healthcare	Site Neuropsychologist
David	Labiner		MD	Arizona Health Sciences Center	Site PI
Hadley	Lange			Minnesota Epilepsy Group	Site Neuropsychologist
Erin	Latif		MD	Augusta University	OB co-Investigator
Connie Lau	Lau		MS	Northwell Health	Research Assistant

Supplemental Online Content: Nonauthor Collaborators

*Indicates required information. Only first name, last name, and suffix will appear in PubMed.

Delmaris	Acosta-Cotte		MA	Augusta University	Site Neuropsychologist
Shari	Lawson		MD	Johns Hopkins University	OB Co-Investigator
Brenda	Leung		BS	The Emmes Company	Programmer Analyst
William	MacAllister		PhD	New York University	Site Neuropsychologist
James	Maciulla		MD	University of Arizona	OB Co-Investigator
Hayley	Madeiros		BS	Boston Children’s Hospital	Site Psychometrist
Nazin	Mahmood		MD	Henry Ford Hospital	Co-Investigator
Jennie	Mao		MD	University of Washington	OB Co-Investigator
Ryan	May		PhD	The Emmes Company	Former Muliti PI
Paul	McCabe		MD	Geisinger Clinic	Retired Site PI
Frederick T.	McElrath		MD	Brigham & Women’s Hospital	Co-Director of OB Core
Erica Meltzer	Meltzer		MD	Northwell Health	Site Neuropsychologist
Lucy	Mendoza		CCRP	University of Cincinnati	Research Assistant
Emily	Miller		MD	Northwestern University	OB Co-Investigator
John W.	Miller		MD	University of Washington	Previous Site PI
Michelle	Miranda		PhD	University of Miami	Site Psychometrist
Jennifer	Moon		PsyD	University of Arizona	Site Neuropsychologist
Eugene	Moore		BS	Emory University	Multisite Research Project Manager
Melissa	Morris		MA	Wake Forest University Health Sciences	Site Neuropsychologist
Chris	Morrison		PhD	New York University	Site Neuropsychologist
Lorene	Nelson		PhD	Stanford University	Consultant and Executive Committee Member
Melanee	Newman		RN	Emory University	Research Assistant
Alisha	Olson		RN	Minnesota Epilepsy Group	Research Assistant
Kim	Ono		PhD	Emory University Children’s Healthcare	Site Neuropsychologist
John	Owen		MD	University of Alabama at Birmingham	OB Co-Investigator
Alison	Pack		MD	Columbia University	
Michael	Paglia		MD	Geisinger Clinic	OB co-Investigator
Yong	Park		MD	Augusta University	OB co-Investigator
Lamar	Parker		MD	Wake Forest University Health Sciences	OB Co-Investigator
Christina	Patterson		MD	University of Pittsburgh	Co-Investigator
Sonia	Perez		PhD	University of Southern California	Site Neuropsychologist
Jenny	Pohlman		MBS	Minnesota Epilepsy Group	Research Assistant
Alison	Pritchard		PhD	John Hopkins University	Site Neuropsychologist
Michael	Privitera		MD	University of Cincinnati	Co-Investigator
Krestin	Radonovich		PhD	University of Pittsburgh	Site Neuropsychologist
Patty	Ray		PhD	Augusta University	Research Assistant
Katie	Reger		PhD	Minnesota Epilepsy Group	Site Neuropsychologist
Gustavo	Rey		PhD	University of Miami	Site Neuropsychologist
Matthew	Ryan		MS	John Hopkins University	Site Neuropsychologist
Yasin	Salih		MD	University of Miami	OB Co-Investigator
Carla	Sandles		CCRP	Henry Ford Hospital	Research Assistant
William	Schweizer		MD	New York University	OB Co-Investigator
Jordan	Seliger		MA	Stanford University	Research Assistant
Enrique	Serrano		MD	University of Miami	Co-Investigator
Nilay	Shah		MD	The Emmes Company	Medical Monitor
Elizabeth	Shashkova		BS	Brigham & Women’s Hospital	Research Assistant
Traci	Sheer		BA	The Emmes Company	Data Manager/Protocol Monitor
Yvonne	Sheldon		RN	Brigham & Women’s Hospital	Neonatal Research Nurse
Rachel	Sierra		RC	University of Southern California	Research Assistant
Marianna	Spanaki-Varelas		MD	Henry Ford Hospital	OB Co-Investigator
Anna	Steele			Minnesota Epilepsy Group	Site Neuropsychologist
Jennifer	Steele		BA	University of Washington	Research Assistant
Alice	Stek		MD	University of Southern California	OB Co-Investigator
Zachary	Stowe		MD	University of Wisconsin	Director of Psychiatric Core
Jolie	Strauss		MA	Boston Children’s Hospital	Site Psychometrist
Suzanne	Strickland		MD	Augusta University	Site PI
Melissa	Sutcliffe		PhD	University of Pittsburgh	Site Neuropsychologist
Hima Bindu Tam	Tam		MD	Northwell Health	OB Co-Investigator
Diane	Teagarden		MSN	Emory University	Co-Investigator
Andrea	Thomas		MS	Henry Ford Hospital	Site Neuropsychologist
Matthew	Thompson		PsyD	University of Alabama at Birmingham	Site Neuropsychologist
Jeffery	Tsai		MD	University of Washington	Site PI
Alexandra	Urban		MD	University of Pittsburgh	Co-Investigator

*Indicates required information. Only first name, last name, and suffix will appear in PubMed.

Delmaris	Acosta-Cotte		MA	Augusta University	Site Neuropsychologist
Linda	Van Marter		MD	John Hopkins University	Director of Neonatal Core
Naymee	Velez-Ruiz		MD	University of Miami	Site PI
Yue	Wang		MS	The Emmes Company	SAS Programmer
Vibhangini	Wasade		MD	Henry Ford Hospital	Co-Investigator
Taylor	Weinau		BS	Brigham & Women's Hospital	OB Research Assistant
Peter	Wells		PharmD	University of Toronto	Consultant and Executive Committee Member
Carrie	Wiles		MS	University of Pittsburgh	Site Neuropsychologist
Mark	Yerby		MD	Oregon Health Sciences University	Consultant and Executive Committee Member
Amy	Young		PsyD	Boston Children's Hospital	Site Neuropsychologist
Andrew	Zillgitt		DO	Henry Ford Hospital	Co-Investigator
Annette	Zygmunt		PhD	Columbia University	Site Neuropsychologist
